# Oligomerization and Nitration of the Grass Pollen Allergen Phl p 5 by Ozone, Nitrogen Dioxide, and Peroxynitrite: Reaction Products, Kinetics, and Health Effects

**DOI:** 10.3390/ijms22147616

**Published:** 2021-07-16

**Authors:** Anna T. Backes, Kathrin Reinmuth-Selzle, Anna Lena Leifke, Kira Ziegler, Carola S. Krevert, Georg Tscheuschner, Kurt Lucas, Michael G. Weller, Thomas Berkemeier, Ulrich Pöschl, Janine Fröhlich-Nowoisky

**Affiliations:** 1 Multiphase Chemistry Department, Max Planck Institute for Chemistry, 55128 Mainz, Germany; k.selzle@mpic.de (K.R.-S.); a.leifke@mpic.de (A.L.L.); kira.ziegler@hotmail.com (K.Z.); krevertc@mpip-mainz.mpg.de (C.S.K.); k.lucas@mpic.de (K.L.); t.berkemeier@mpic.de (T.B.); u.poschl@mpic.de (U.P.); 2 Division 1.5 Protein Analysis, Federal Institute for Materials Research and Testing (BAM), 12489 Berlin, Germany; georg.tscheuschner@bam.de (G.T.); michael.weller@bam.de (M.G.W.)

**Keywords:** tyrosine, nitrotyrosine, dityrosine, nitration degree, protein dimer, protein oligomer

## Abstract

The allergenic and inflammatory potential of proteins can be enhanced by chemical modification upon exposure to atmospheric or physiological oxidants. The molecular mechanisms and kinetics of such modifications, however, have not yet been fully resolved. We investigated the oligomerization and nitration of the grass pollen allergen Phl p 5 by ozone (O_3_), nitrogen dioxide (NO_2_), and peroxynitrite (ONOO^–^). Within several hours of exposure to atmospherically relevant concentration levels of O_3_ and NO_2_, up to 50% of Phl p 5 were converted into protein oligomers, likely by formation of dityrosine cross-links. Assuming that tyrosine residues are the preferential site of nitration, up to 10% of the 12 tyrosine residues per protein monomer were nitrated. For the reaction with peroxynitrite, the largest oligomer mass fractions (up to 50%) were found for equimolar concentrations of peroxynitrite over tyrosine residues. With excess peroxynitrite, the nitration degrees increased up to 40% whereas the oligomer mass fractions decreased to 20%. Our results suggest that protein oligomerization and nitration are competing processes, which is consistent with a two-step mechanism involving a reactive oxygen intermediate (ROI), as observed for other proteins. The modified proteins can promote pro-inflammatory cellular signaling that may contribute to chronic inflammation and allergies in response to air pollution.

## 1. Introduction

The prevalence of allergic diseases and asthma is increasing worldwide, and especially the complexity and severity in children and young adults continue to rise, particularly in newly industrialized countries such as China or India [1,2,3,4,5]. The rapid increase in the prevalence, however, cannot be explained by genetic factors alone, and environmental factors must be taken into account [6,7,8,9]. Indeed, several studies have suggested that allergic diseases are enhanced by traffic-related air pollution [10,11,12], and it has been shown that birch and ragweed pollen from urban areas had a higher allergenic potential than pollen from rural areas [13,14]. Pollen can rupture upon exposure to anthropogenic air pollutants such as ozone (O_3_) or nitrogen dioxide (NO_2_) or under humid conditions and release cytoplasmic material including allergenic proteins and biogenic adjuvants into the environment [15,16,17,18]. The released proteins can attach to larger particles such as soil or road dust or be dissolved in water, where they can interact in an aqueous environment with gaseous and particulate air pollutants [19,20,21]. The results are chemical posttranslational modifications, which can change the protein structure and stability, affect polarity and acidity of binding sites, and therefore alter the immunogenicity of the proteins [22,23,24,25,26,27].

In the atmosphere, proteins can react with reactive oxygen and nitrogen species (ROS, RNS) such as O_3_ and NO_2_, resulting in oxidized, nitrated, and degraded proteins as well as protein oligomers [19,21,28]. In addition to chemical modification in the environment, inhaled air pollutants can lead to ROS production, oxidative stress, and inflammation in the human body [29,30,31]. During these processes, nitrogen oxide radicals (NO) and superoxide anions (O_2_^–^) are formed, which rapidly react to peroxynitrite (ONOO^–^) [32]. Similar to the reaction of proteins with O_3_ and NO_2_, peroxynitrite can also lead to protein oligomerization and nitration [33,34,35]. Although several studies were already published investigating the reaction mechanisms and kinetics of chemical protein modification, they do not allow drawing conclusions regarding possible health effects of modified proteins. To investigate real health effects, studies with modified health-related proteins such as allergens or other immunomodulatory proteins are indispensable.

In this study, we investigated the chemical modification of recombinant Phl p 5.0101, simply termed Phl p 5 hereafter, a major allergen of timothy grass pollen (*Phleum pratense*). Exposure to Phl p 5 can induce rhinitis and bronchial asthma in patients allergic to grass pollen [36]. The native protein function, however, is not yet clear, but it is supposed, that the protein plays a role in pollen germination [37]. The protein is estimated to represent about 6% (*w*/*w*) of the whole pollen extract [38]. The recombinant protein has a molecular weight of 28.6 kDa according to the manufacturer and consists of 287 amino acids including 12 tyrosine residues (∼4%). The structure of the protein comprises two flexibly connected domains of similar size that include identical secondary structural elements such as four parallel α-helices [37].

To mimic air pollution effects, recombinant Phl p 5 was exposed to a mixture of O_3_ and NO_2_ in aqueous phase under various atmospherically relevant conditions (O_3_/NO_2_ concentrations: 50 ppb/50 ppb, 200 ppb/200 ppb; exposure time: 0.5–10 h). Please note that the experimental design used in this study (small gas bubbles in a large volume of liquid) is only partially comparable to exposure in the atmosphere (small liquid droplets in a large volume of gas). In both systems, however, the rate of protein modification is likely determined by gas solubility and chemical reaction rate. In contrast, under dry conditions or in a (semi-)solid aerosol matrix, slow diffusion may influence reaction kinetics and lead to different oligomerization and nitration degrees [39]. For the investigation of endogenous protein modification during oxidative stress, Phl p 5 was treated with different ONOO^–^ concentrations (0.04–2 mM). Size-exclusion chromatography was used to analyze protein oligomer mass fractions and their tyrosine nitration degrees simultaneously, and the different protein fractions were confirmed by SDS-PAGE. Reversed-phase chromatography was used to determine the total tyrosine nitration degree and estimate the number of nitrotyrosine residues. For selected samples, the results were compared to MALDI-TOF-MS measurements. To our knowledge, this is the first time that the recombinant grass pollen allergen Phl p 5 has been modified by ozone and nitrogen dioxide as well as peroxynitrite to mimic exogenous and endogenous protein modifications through direct and indirect air pollution effects.

## 2. Results and Discussion

### 2.1. Oligomerization

Native and modified Phl p 5 samples were analyzed by size-exclusion chromatography (SEC-HPLC) for protein dimers and higher oligomers (for exemplary chromatograms, see Supplementary Materials Figure S1). Native Phl p 5 consists primarily of protein monomers (>99%), but also contains a small mass fraction of dimers (<1%). For native Phl p 5, higher oligomers (MW > MW_dimer_) were not detectable in our study.

**Figure 1:**
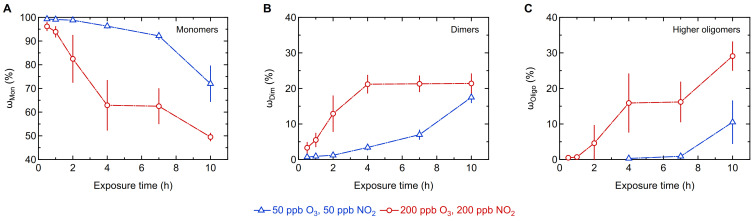
Oligomerization of Phl p 5 exposed to low (blue triangles) and high (red circles) concentrations of ozone (O_3_) and nitrogen dioxide (NO_2_) for different exposure times determined by size-exclusion chromatography (SEC-HPLC): Mass fractions of (**A**) monomers (ω_Mon_), (**B**) dimers (ω_Dim_), (**C**) higher oligomers (MW > MW_dimer_; ω_Oligo_). Arithmetic mean values and standard deviations of two to five independent experiments measured in duplicates, respectively. Lines are to guide the eye.

Figure 1 shows the effect of low (50 ppb/50 ppb) and high (200 ppb/200 ppb) concentrations of O_3_/NO_2_ on the oligomerization of Phl p 5 for different exposure times. Generally, the mass fractions of Phl p 5 dimers and higher oligomers increased over the course of exposure time while the mass fraction of monomers decreased. We found up to 18 ± 2% dimers at low O_3_/NO_2_ concentrations and up to 21 ± 3% at high oxidant concentrations after 10 h of exposure (Figure 1B). While the final dimer mass fractions were similar, the temporal evolution of the dimer mass fractions deviated for the two exposure levels. At low O_3_/NO_2_ concentrations, the dimer mass fraction increased only slightly at short exposure times, but showed a steeper increase for longer exposure times. In contrast, at high oxidant concentrations, the dimer mass fraction increased rapidly within the first 4 h, but stayed constant at longer exposure times.

The mass fraction of higher oligomers (MW > MW_dimer_) showed a strong and gradual increase over time at high O_3_/NO_2_ concentrations. At low oxidant concentrations, however, the detection of higher oligomers was not possible at short exposure times, and a significant increase is only shown for long exposure times reaching 11 ± 6% after 10 h of exposure (Figure 1C). The largest higher oligomer fraction (29 ± 4%) was found for exposure to high O_3_/NO_2_ concentrations for 10 h. The mass fraction of higher oligomers thus exceeds the dimer mass fraction at high oxidant concentrations and long exposure times. The corresponding mass fraction of Phl p 5 monomers decreased significantly over the course of exposure time, and the minimum Phl p 5 monomer mass fraction found in this study was 72 ± 8% for low O_3_/NO_2_ concentrations and 50 ± 2% for high oxidant concentrations (Figure 1A). As the monomer mass fraction continued to decline and the higher oligomer mass fraction continued to increase up to the 10 h mark, we expect an even larger fraction of higher oligomers for longer exposure times than those investigated in this study, while the dimer mass fraction will remain in a dynamic equilibrium of formation and consumption as long as protein monomers are available.

To investigate the effect of exposure to the single oxidants, we performed additional experiments using only O_3_ or only NO_2_. For the exposure to 200 ppb O_3_ for 2 h, we found 20± 1% of dimers and 15± 3% of higher oligomers, which are both higher than those found for exposure to the mixture of 200 ppb O_3_ and 200 ppb NO_2_ over the same time. This could be explained by the underlying reaction mechanism, which was hypothesized previously to consist of two steps [40,41,42]. According to this mechanism, a tyrosine residue of the protein first reacts with O_3_ to form a tyrosyl radical as long-lived reactive oxygen intermediate (ROI) (R1). Due to their long lifetime (>10 min [42]), ROI may react in a second step either with NO_2_, resulting in the formation of 3-nitrotyrosine (NTyr) residues (R2), combine with other ROI forming dityrosine (DiTyr) cross-links (R3), or undergo further oxidation reactions. Our findings of lower oligomer mass fractions upon exposure to the mixture of O_3_/NO_2_ could be explained by a competition between NO_2_ and a second tyrosyl radical in the second reaction step once the ROI is formed.

(R1) $$\mathrm{Tyr}+O_{3}\to \mathrm{ROI}$$

(R2) $$\mathrm{ROI}+{\mathrm{NO}}_{2}\to \mathrm{NTyr}$$

(R3) ROI+ROI→DiTyr

We also tested the effect of exposure to 200 ppb NO_2_ for 2 h without the addition of O_3_ and found a dimer mass fraction of 6± 1%, but no higher oligomers. This observation is unexpected as the reaction mechanism described above requires O_3_ for ROI formation [42]. Our findings show that ROI may also be formed by NO_2_, though at a lower rate (R4) [19,43]. The so formed ROI can then undergo further reactions as already described above (R2, R3).

(R4) $$\mathrm{Tyr}+{\mathrm{NO}}_{2}\to \mathrm{ROI}$$

A lower steady-state concentration of ROI compared to the mixed O_3_/NO_2_ experiments and the presence of sufficient NO_2_ leads to less formation of dimers, in line with the original mechanism. Alternatively, also other possible cross-linking site reactions such as e.g., the formation of protein carbonyl–lysin schiff bases may occur [44,45].

**Figure 2:**
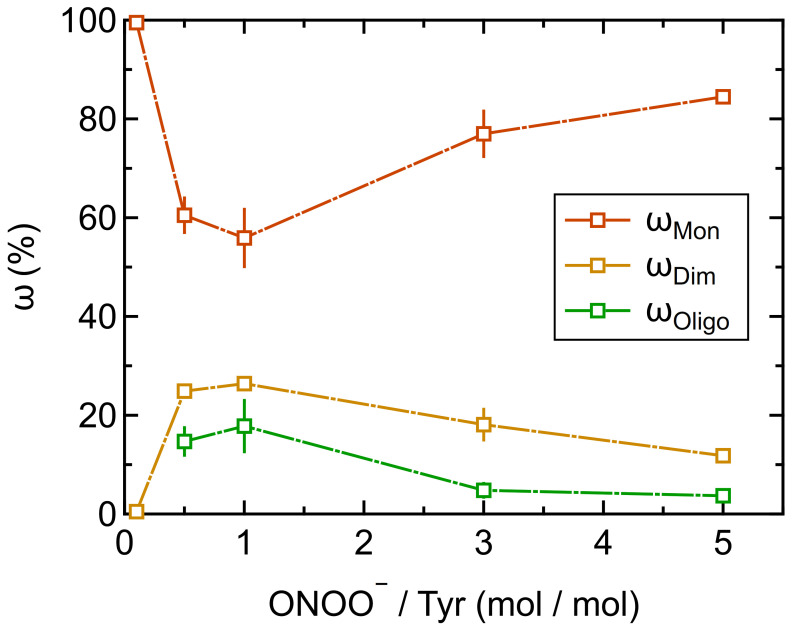
Protein oligomer mass fractions of Phl p 5 treated with different molar ratios of peroxynitrite over tyrosine residues (ONOO^–^/Tyr) determined by SEC-HPLC: monomers (ω_Mon_), dimers (ω_Dim_) and higher oligomers (MW > MW_dimer_; ω_Oligo_). Arithmetic mean values and standard deviations of two independent experiments measured in duplicates. Lines are to guide the eye.

Figure 2 shows the effect of different concentrations of ONOO^–^ on the oligomerization of Phl p 5. The largest oligomer mass fractions were found for equimolar concentrations of peroxynitrite and Tyr residues (ONOO^–^/Tyr: 1/1), reaching up to 26 ± 1% for dimers and 18 ± 5% for higher oligomers. The corresponding minimum mass fraction of monomers was 56 ± 6%. For higher molar ratios of ONOO^–^/Tyr, however, lower oligomer mass fractions were found.

The formation of a dityrosine cross-link between two protein monomers by peroxynitrite is thought to be a two-step mechanism, in which first two tyrosyl radicals form that subsequently combine [34]. Ammonium bicarbonate buffer ensures alkaline reaction conditions and triggers the reaction of ONOO^–^ with CO_2_ forming nitrosoperoxycarbonate (ONOOCO_2_^–^), which rapidly decomposes to NO_2_ and a carbonate radical (CO_3_^–^) [46,47]. Both radicals may react with the protein to form a tyrosyl radical; however, radical formation by CO_3_^–^ is likely much faster and may thus be the dominant reaction pathway at excess concentrations of ONOO^–^. This scenario would lead to both high tyrosyl radical and high NO_2_ concentrations and hence favor protein nitration, which will be discussed below [48]. At low ONOO^–^ concentrations, however, additional tyrosyl radical formation by NO_2_ would lead to tyrosyl radical formation at a comparatively low NO_2_ concentration, which would favor the formation of dimers and higher oligomers, in accordance with our observations. This is also in good agreement with Pfeiffer et al. [34], who found that the dimerization of Tyr radicals outcompeted the nitration reaction at low ONOO^–^ concentrations.

**Figure 3:**
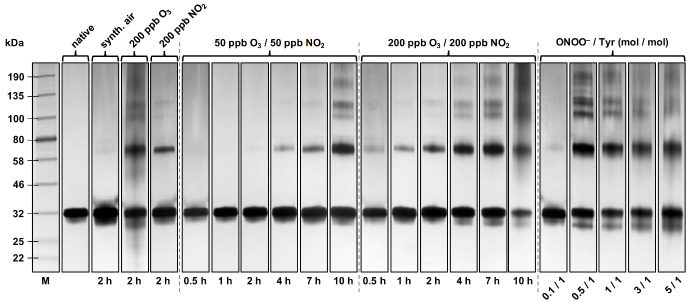
SDS-PAGE with silver stain for Phl p 5 before and after exposure to O_3_/NO_2_ and reaction with ONOO^–^, respectively. For each lane, 50 ng Phl p 5 was loaded onto the gel. Expected molecular masses: monomer (28.6 kDa), dimer (57.2 kDa), trimer (85.8 kDa), and tetramer (114.4 kDa).

SDS-PAGE analysis with silver stain of Phl p 5 before and after exposure to O_3_/NO_2_ and reaction with ONOO^–^, respectively, was performed to confirm and visualize the size-exclusion chromatography results. The expected molecular masses of the Phl p 5 protein fractions are 28.6 kDa for monomers, 57.2 kDa for dimers, 85.8 kDa for trimers, and 114.4 kDa for tetramers. Figure 3 shows that native Phl p 5 mainly consists of monomers, and that these monomers were cross-linked to dimers and higher oligomers dependent on the reagent, its concentration, and exposure time.

A comparison of both experiments, exposure to O_3_/NO_2_ and reaction with ONOO^–^, reveals that significant formation of protein dimers and higher oligomers is achieved with either method. The total and relative amounts, however, depend on the exact reaction conditions. While the maximum dimer and higher oligomer mass fractions for the reaction with ONOO^–^ were found at equimolar concentrations of ONOO^–^ and tyrosine residues, we assume to find an even larger mass fraction of higher oligomers for exposure to O_3_/NO_2_ at longer exposure times than investigated in this study. It has to be taken into account that the exposure to O_3_/NO_2_ occurred in pure water, whereas for the reaction with ONOO^–^, the protein was further stabilized in a buffer solution. In pure water, the protein is directly exposed to the highly reactive gases O_3_ and NO_2_, that could additionally lead to protein denaturation and unfolding. Ke and Huang [49] reported that the protein structure and conformation can influence the efficiency of dityrosine cross-linking as they found that proteins in an unstructured state can be more easily cross-linked via dityrosine formation. Thus, partial unfolding of Phl p 5 in pure water could in part explain the high oligomerization degrees observed for the exposure to O_3_/NO_2_ in this study and may lead to sustained oligomerization at even longer exposure times.

### 2.2. Nitration

Native and modified Phl p 5 samples were analyzed by reversed-phase chromatography (RP-HPLC) for their total tyrosine nitration degree, and the number of nitrotyrosine residues was calculated. For comparison, MALDI-TOF-MS experiments were performed with selected samples (for spectra, see Supplementary Materials Figure S2). Native Phl p 5 has a total tyrosine nitration degree (ND_tot_) of 0.3%, but all results of modified proteins shown in this study were not corrected for this value.

**Figure 4:**
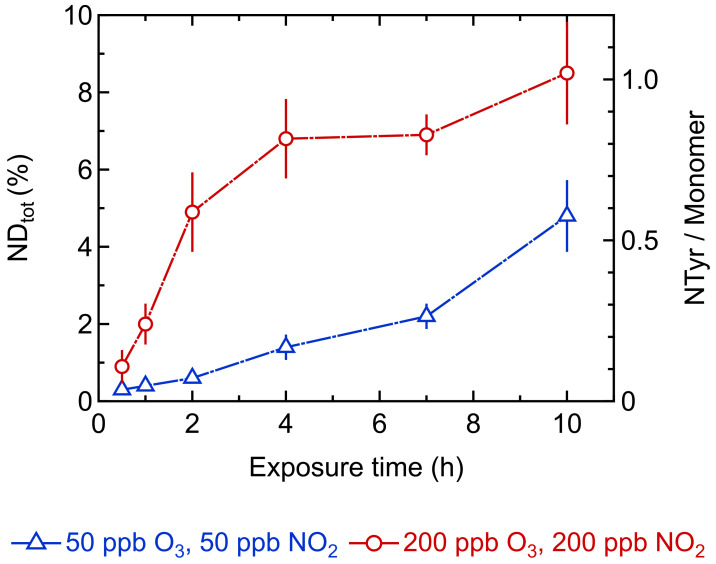
Total nitration degree (ND${}_{\mathrm{tot}}$) of Phl p 5 exposed to low (blue triangles) and high (red circles) concentrations of O_3_/NO_2_ determined by reversed-phase chromatography (RP-HPLC) (primary y-axis) and average number of nitrotyrosine (NTyr) residues calculated per Phl p 5 monomer (secondary y-axis) for different exposure times. Arithmetic mean values and standard deviations of two to five independent experiments measured in duplicates. Lines are to guide the eye.

Figure 4 shows the total tyrosine nitration degree of Phl p 5 at low (50 ppb/50 ppb) and high (200 ppb/200 ppb) concentrations of O_3_ and NO_2_, determined by UV absorbance. ND_tot_ increased over the course of exposure time and with increasing O_3_/NO_2_ concentrations. For high oxidant concentrations, the curve runs asymptotically towards a maximum ND_tot_ of 9 ± 1%, which was reached after 10 h of exposure. Considering 12 Tyr residues per Phl p 5 molecule, the maximum ND_tot_ corresponds to an average of approx. one nitrotyrosine (NTyr) residue calculated per Phl p 5 monomer. Exposure at low O_3_/NO_2_ concentrations shows a gradual increase with exposure time, reaching a ND_tot_ of 5 ± 1% after 10 h. From extrapolation, we assume that at low oxidant concentrations, longer exposure times are needed to reach a similar maximum ND_tot_ as found for exposure at high oxidant concentrations.

These results are in good agreement with our previous study on the chemical modification of the major birch pollen allergen Bet v 1.0101 by O_3_/NO_2_ in aqueous phase [28]. That study reported a nitration degree of ∼22 ±7% after 17 h of exposure at 120 ppb O_3_ and 120 ppb NO_2_. The reaction conditions are comparable to the ones used in this study (in total ∼2 ppm h of each gas), so that the absolute nitration degree was higher for Bet v 1 compared to Phl p 5. Considering that the Bet v 1 molecule contains in total only 7 Tyr residues, the relative nitration degree per tyrosine residue is similar to the results obtained in this study. For both allergens, approx. one tyrosine residue was nitrated under the investigated conditions.

**Figure 5:**
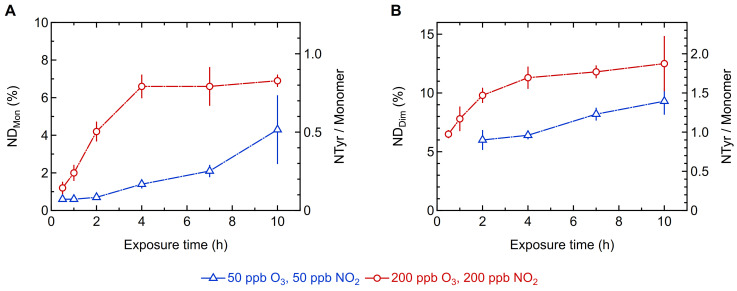
Nitration degrees of different Phl p 5 protein mass fractions determined by SEC-HPLC (primary y-axis) and average number of nitrotyrosine (NTyr) residues calculated per Phl p 5 monomer (secondary y-axis) for different exposure times: (**A**) monomers (ND_Mon_), (**B**) dimers (ND_Dim_). Arithmetic mean values and standard deviations of two to five independent experiments measured in duplicates. Lines are to guide the eye.

Figure 5 shows that the nitration degrees of protein monomers and dimers also increased gradually over the course of exposure time. The maximum nitration degree of monomers was 4 ± 2% for low O_3_/NO_2_ concentrations and 7 ± 1% for high oxidant concentrations after 10 h of exposure (Figure 5A), which is consistent with the results obtained by RP-HPLC for the total nitration degree. The maximum nitration degree for the Phl p 5 dimers was 9 ± 1% for low O_3_/NO_2_ concentrations and 13 ± 2% for high oxidant concentrations after 10 h of exposure (Figure 5B). For the higher oligomers (MW > MW_dimer_), the nitration degree could not be reliably quantified due to the low concentrations of the protein samples. Interestingly, the dimers seem to exhibit a higher nitration degree than the monomers. However, it cannot be fully excluded that other protein modifications such as dityrosine and hydroxytyrosine affect the determination of the nitration degree due to interference with the absorption spectrum of tyrosine and nitrotyrosine [50]. This hypothesis is supported by the results of the pure O_3_ exposure experiment, in which a total nitration degree of 3.5% was found, although no NO_2_ was applied. In this case, the formation of nitrotyrosine can be excluded and other modifications of the tyrosine residue must have inferred with the absorption spectrum and led to a slight positive bias in the nitration degree. It can thus not be determined with certainty that dimers are more strongly nitrated than monomers.

**Figure 6:**
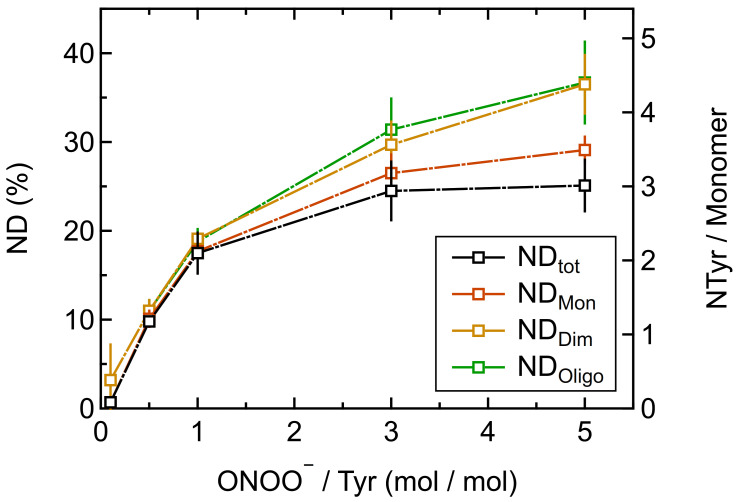
Total nitration degree determined by RP-HPLC and nitration degrees of different Phl p 5 protein mass fractions determined by SEC-HPLC (primary y-axis) as well as number of nitrotyrosine (NTyr) residues calculated per Phl p 5 monomer (secondary y-axis) for different molar ratios of peroxynitrite over tyrosine residues (ONOO^–^/Tyr). Arithmetic mean values and standard deviations of two to three independent experiments measured in duplicates. Lines are to guide the eye.

Figure 6 shows the total tyrosine nitration degree for the reaction of peroxynitrite with Phl p 5. Increasing the molar ratio of peroxynitrite over tyrosine (ONOO^–^/Tyr) resulted in an increasing ND_tot_. The curve runs asymptotically towards a maximum ND_tot_ of 25 ± 3%, which was reached for the ONOO^–^/Tyr molar ratio of 5/1. Considering 12 Tyr residues per Phl p 5 molecule, the maximum ND_tot_ corresponds to an average of ∼3 NTyr residues calculated per Phl p 5 monomer.

The tyrosine nitration degrees for Phl p 5 monomers, dimers, and higher oligomers increased with increasing molar ratio of peroxynitrite over tyrosine (ONOO^–^/Tyr) similar to ND_tot_ (Figure 6). The maximum nitration degree for the Phl p 5 monomers was 29 ± 2%, which corresponds to an average of ∼3.5 NTyr residues calculated per Phl p 5 monomer. The maximum nitration degree for the Phl p 5 dimers was 37 ± 3%, which is higher than the monomers and corresponds to an average of ∼4.5 NTyr residues calculated per Phl p 5 monomer. The maximum nitration degree for the higher oligomers (MW > MW_dimer_) was 37 ± 5% and similar to the dimers. The nitration degrees for monomers, dimers, and higher oligomers appear to be higher than the total nitration degree. Here, it needs to be considered that the nitration degrees were determined by two different methods (ND_tot_: RP-HPLC at pH 3.5; ND_Mon_, ND_Dim_, ND_Oligo_: SEC-HPLC at pH 7). This pH change can affect the absorption spectrum of the modified amino acid residues [51,52]. Please note that for nitrotyrosine, the pH dependency of the NTyr extinction coefficient was already taken into consideration for both methods [53,54], but it might need to be considered for other modified amino acids as well.

In addition, the nitration results of the two protein modification methods were confirmed by MALDI measurements, exemplarily for selected samples (Table 1). The good agreement of results from UV absorbance and mass spectrometry suggests that primarily nitrotyrosine and no or only little other oxidation products were formed. The nitration and oxidation of other amino acids such as phenylalanine, however, cannot be excluded, but is rather unlikely based on previous studies [28,55,56].

Both exposure to O_3_/NO_2_ and reaction with ONOO^–^ led to the formation of nitrotyrosine and thus to protein nitration. Comparing both protein modification pathways, we can conclude that under the conditions used in this study, the highest nitration degrees were found for the reaction with ONOO^–^. While at most only one tyrosine residue per Phl p 5 monomer could be nitrated upon exposure to O_3_/NO_2_, approximately three nitrotyrosine residues per Phl p 5 monomer were found for the reaction with ONOO^–^. Moreover, other reactant concentrations, the different reaction media could play a role here. In the reaction with ONOO^–^, the proteins are stabilized in a buffer solution, but exposure to O_3_/NO_2_ occurs in pure water. The modifications of Phl p 5 could lead to conformational changes of the protein, which could result in an altered accessibility of the individual tyrosine residues for the different reagents. Moreover, the concentration of ONOO^–^ (2 mM) is much larger than the amount of dissolved NO_2_ from bubbling (∼2 nM using a Henry’s law coefficient of ∼10^−2^ M atm^−1^ [57]), which may lead to a higher NO_2_ radical concentration in the experiments using ONOO^–^. However, without proper knowledge of the ONOO^–^ decomposition kinetics, the in situ NO_2_ concentrations in aqueous solution could not be determined.

### 2.3. Comparison Oligomerization Vs. Nitration

**Figure 7:**
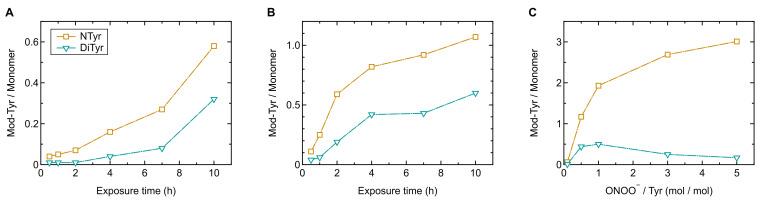
Nitrated and cross-linked tyrosine residues calculated per Phl p 5 monomer after exposure to low (**A**) and high (**B**) O_3_/NO_2_ concentrations and reaction with ONOO^–^ (**C**). Lines are to guide the eye.

For a better comparison of protein oligomerization and nitration in this study, but also with other proteins from related studies, we calculated how many tyrosine residues on average were cross-linked in dityrosines. The so-called dityrosine degree (DD) enables the direct comparison of modifications on tyrosine level and thus takes into account the total amount of tyrosine residues in a protein. Table 1 shows the dityrosine degree and the average number of tyrosine residues, which were cross-linked in dityrosines, calculated per Phl p 5 monomer in comparison to the nitration results for selected samples. Evidently, protein nitration occurs more readily than protein oligomerization as nitration degrees are larger than dityrosine degrees for all investigated samples. Figure 7 shows the temporal evolution of nitrated and cross-linked tyrosine residues for the samples, which were exposed to low (A) and high (B) concentrations of O_3_/NO_2_, and the concentration dependency of nitrated and cross-linked tyrosine residues for the reaction with ONOO^–^ (**C**). The direct comparison of nitrated tyrosine residues and those cross-linked in dityrosines shows that more tyrosine residues were nitrated than cross-linked under the same conditions. Considering the reaction mechanisms for both exposure to O_3_/NO_2_ and reaction with ONOO^–^, this is not that surprising, as in the competition for the ROI, the NO_2_ radical is sterically at an advantage compared to the rather bulky secondary ROI. Furthermore, there should be much more NO_2_ radicals than ROI in the reaction mixture, so that the reaction of ROI with NO_2_ is more likely compared to the combination of two ROI. This hypothesis is in agreement with an earlier study of the model protein bovine serum albumin (BSA), which found that protein nitration is favored over protein oligomerization [41]. The influence of protein concentration on the oligomerization and nitration, however, will be investigated in future studies.

The concentrations of O_3_ and NO_2_ used in this study are close to levels typically encountered in the atmosphere. The world health organization (WHO) set the guideline value for ozone levels to 100 µg m^−3^ (approx. 50 ppb) for an 8-hour daily average to provide adequate protection of public health, while the European union transposed the directive to a limit of 120 µg m^−3^. In Germany, the annual mean was on average 51 µg m^−3^ for the years 1995 to 2019, and the EU level was exceeded on average on 22 days per year in this time period [58,59]. The WHO guideline value for nitrogen dioxide was set to 200 µg m^−3^ (approx. 100 ppb) for an 1-h mean and 40 µg m^−3^ for an annual mean. In Germany, the NO_2_ emissions are decreasing over recent decades, and they were on average 25 µg m^−3^ in 2019, but 12% of the measurement stations exceeded the limit of the annual mean [60].

The peroxynitrite concentrations in the human body, however, are difficult to determine precisely, as the strong oxidizing and nitrating reagent is formed in vivo during oxidative stress and inflammation from nitrogen oxide radicals (NO) and superoxide anions (O_2_^–^) [32]. Kouti et al. [61] found peroxynitrite serum levels of ∼7 µmol L^−1^ in patients with Parkinson’s disease and ∼4 µmol L^−1^ in the control group, which is less than we used in our study. It cannot be excluded, however, that higher peroxynitrite concentrations might occur at inflammation sites.

The products of the reactions of O_3_, NO_2_, and ONOO^–^ with Phl p 5 include various nitrated and cross-linked proteins, all of which might have a different allergenic potential. Until now, most studies focused on protein nitration as a potential enhancer for allergic and other severe diseases (e.g., [19,26,27]), but also other modifications such as protein cross-linking need to be considered. Thus, further studies are required to investigate the immunogenicity of the different nitrated and cross-linked variants, to complement the information on reaction kinetics obtained in this study. Furthermore, realistic and relevant mixtures of nitrated and cross-linked proteins are necessary to assess the whole health-related potential of modified proteins and elucidate their role in modulated immune responses. The efficient oligomerization and nitration of allergenic proteins by air pollutants and the enhanced allergenic and immunostimulatory potential of chemically modified proteins call for action to improve air quality and public health in the Anthropocene.

## 3. Materials and Methods

Pure water was taken from a Barnstead™ GenPure™ xCAD plus water purification system (Thermo Scientific, Braunschweig, Germany). The water was autoclaved at 121 ${}^{\circ }$C for 20 min and filtered three times through a sterile 0.1 µm pore diameter polyethersulfone (PES) vacuum filter unit (VWR International, Radnor, PA, USA). Recombinant timothy grass (*Phleum pratense*) pollen allergen 5 (Phl p 5.0101) was obtained from Biomay AG (Vienna, Austria), and a stock solution (1 mg mL^−1^) was prepared with pure water prior to each experiment. The solution was reconstituted at room temperature for at least 30 min to ensure complete reconstitution of the protein. Sodium peroxynitrite (160–200 mM) was purchased from Merck (Darmstadt, Germany), and ammonium bicarbonate (NH_4_HCO_3_), acetonitrile (ACN), Tris-HCl, Glycerol, SDS, and monobasic sodium phosphate monohydrate (NaH_2_PO_4_· H_2_O) were from Carl Roth GmbH & Co. KG (Karlsruhe, Germany). A 150 mM NaH_2_PO_4_· H_2_O buffer was prepared, and the pH was adjusted to 7 by the addition of sodium hydroxide (NaOH). NaOH and water with 0.1% trifluoroacetic acid (TFA) were obtained from VWR International GmbH (Darmstadt, Germany). α-Cyano-4-hydroxycinnamic acid (HCCA, ultrapure, J67635) was purchased from Th. Geyer GmbH & Co. KG (Renningen, Germany). Trifluoroacetic acid (TFA, LC-MS grade, 85183) was obtained from Thermo Fisher Scientific (Braunschweig, Germany).

### 3.1. Protein Modification by O_3_/NO_2_

The experimental setup was described previously [41], and it was extended by incorporating an oven (Heratherm IGS60, Thermo Fisher Scientific) to maintain a constant temperature of 25 ${}^{\circ }$C during the exposure. A schematic of the experimental setup is available in the Supplementary Materials Figure S3. Briefly, O_3_ was generated by synthetic air passing through a UV lamp (L.O.T.-Oriel GmbH & Co. KG, Darmstadt, Germany) at ∼1.9 L min^−1^, and the O_3_ concentration was adjusted by tuning the amount of UV light. The gas flow was humidified by passing through a Nafion^®^ gas humidifier (MH-110-12F-4, PermaPure, Lakewood, NJ, USA) operated with pure water. The gas flow was mixed with N_2_ containing ∼5 ppmV NO_2_ (Air Liquide Deutschland GmbH, Düsseldorf, Germany), and the NO_2_ concentration was regulated by the amount of the added ∼5 ppmV NO_2_ gas. After mixing, the gas flow was split, and one flow was used to react with the samples, and the other one was used to determine the concentrations of O_3_ and NO_2_. For sample exposure, the O_3_/NO_2_ gas mixture was directly bubbled through 1.5 mL of 0.13 mg mL^−1^ Phl p 5 aqueous solution at a flow rate of 60 mL min^−1^ using a Teflon tube (ID: 1.59 mm). exposure times were 0.5, 1, 2, 4, 7, and 10 h, respectively. The O_3_ and NO_2_ concentrations were monitored using commercial monitoring instruments (ozone analyzer: 49i, Thermo Scientific, Braunschweig, Germany; NOx analyzer: T200UP, Teledyne API, San Diego, CA, USA).

After exposure, the samples were concentrated using a 10 kDa centrifugal filter (Amicon^®^ Ultra; Merck). For this, the reaction solution was pipetted into the filter unit and centrifuged at 14,000× *g* for 2 min (5427 R, Eppendorf). The sample was washed five times with 200 µL pure water and centrifuged at 14,000× *g* for 2 min. For sample recovery, the filter was turned upside down into a clean micro centrifuge tube and centrifuged at 1000× *g* for 2 min. To remove possible sample residues, the filter was washed with 100 µL pure water and centrifuged again upside down into the concentrated protein sample. Two to five independent experiments were performed.

### 3.2. Protein Modification by ONOO^–^

For each reaction, 300 µL Phl p 5 protein stock solution (1 mg mL^−1^) was transferred into a brown reaction tube (Eppendorf, Hamburg, Germany), and 7.7 µL NH_4_HCO_3_ buffer (2 M) was added to yield a final buffer concentration of 0.05 M. Phl p 5 was treated with different peroxynitrite concentrations (0.04–2 mM), which were applied in relation to the tyrosine content of the protein (ONOO^–^/Tyr molar ratios: 0.1/1, 0.5/1, 1/1, 3/1, 5/1). After being thawed on ice, an aliquot of peroxynitrite was diluted 1:2 with 0.3 M NaOH to reduce the pipetting error for small volumes. The diluted peroxynitrite solution was added to the protein sample to yield ONOO^–^/Tyr molar ratios of 0.1/1 and 0.5/1, and the original peroxynitrite solution was added to yield ONOO^–^/Tyr molar ratios of 1/1, 3/1, and 5/1. The reaction was performed on ice for 110 min. Afterwards, the sample was purified and concentrated using a 10 kDa centrifugal filter as described in Section 3.1. Three independent experiments were performed.

### 3.3. HPLC-DAD Analysis

The protein oligomer mass fractions and their individual nitration degrees were determined simultaneously as previously described in Liu et al. [53] using a high-performance liquid chromatography system coupled to diode array detection (HPLC-DAD; Agilent Technologies 1260 Infinity II series), which consisted of a binary pump (G7112B), a multisampler (G7167A), a column thermostat (G7116A), and a photodiode array detector (G7115A) monitoring 220, 280, and 357 nm. ChemStation software (Rev. C.01.08, Agilent) was used for system control and data analysis. Molecular weight separation by size-exclusion chromatography (SEC-HPLC) was carried out using a SEC column (PSS Proteema BioInert Micro 300 Å, 250 mm x 4.6 mm inner diameter, 3 µm particle size; PSS Polymer Standards Service GmbH, Mainz, Germany). Isocratic separation was performed at a flow rate of 0.35 mL min^−1^ with 150 mM NaH_2_PO_4_ buffer (pH 7). The sample injection volume was 30 µL, and each chromatographic run was performed in duplicate.To calculate the number of tyrosine residues that are part of dityrosine cross-links, it was assumed for simplification that all higher oligomers are trimers. The protein mass fractions were divided by 100, and the result of the higher oligomer mass fraction was multiplied by 4/3 and added to the result of the dimer mass fraction to obtain the average number of cross-linked tyrosine (DiTyr) residues calculated per Phl p 5 monomer. The dityrosine degree (DD) was calculated by dividing the DiTyr number by 12, as each Phl p 5 monomer contains 12 tyrosine residues, and multiplying by 100.

The total tyrosine nitration degree was determined as described in Selzle et al. [54]. Briefly, a HPLC-DAD system (Agilent Technologies 1260 Infinity series, Waldbronn, Germany) was used, which consisted of a quaternary pump (G1311B), an autosampler (G7129A), a column thermostat (G1316C), and a photodiode array detector (G1315C) monitoring 220, 280, and 357 nm. ChemStation software (Rev. C.01.07, Agilent) was used for system control and data analysis. A monomerically bound C_18_ column (Vydac 238TP, 250 mm × 2.1 mm i.d., 5 µm, Hichrom, Berkshire, UK) was used for reversed-phase chromatography (RP-HPLC). Gradient elution was performed at a flow rate of 0.2 mL min^−1^ with 0.1% (*v*/*v*) TFA in water and ACN. The sample injection volume was 10 µL, and each chromatographic run was performed in duplicate. To calculate the average number of nitrotyrosine (NTyr) residues per Phl p 5 monomer, the total nitration degree was multiplied by 12, which is the number of tyrosine residues per Phl p 5 monomer.

### 3.4. MALDI-TOF-MS Analysis

MALDI-TOF-MS was performed on a Bruker Autoflex maX^TM^ in linear mode. α-Cyano-4-hydroxycinnamic acid (15 mg mL^−1^ in 50% H_2_O, 49.9% ACN, 0.1% TFA (*v*/*v*/*v*)) was used as the matrix. Samples were spotted using the dried-droplet method with 1 µL of sample solution and 1 µL of matrix solution. The obtained spectra were averaged over 5000 laser shots. The average number of nitrotyrosine (NTyr) residues per Phl p 5 monomer was calculated as nitro groups from the peak shift between native and modified sample.

### 3.5. SDS-PAGE and Silver Stain

Native and modified Phl p 5 samples were mixed with an equivalent volume of 2× Laemmli buffer, containing 65.8 mM Tris-HCl (pH 6.8), 26.3% glycerol (*v*/*v*), 2.1% SDS and 0.01% bromophenol blue, and heated at 95 ${}^{\circ }$C for 5 min. 50 ng per sample were loaded onto a PROTEAN Precast gel (4–20%, Bio-Rad, Munich, Germany) together with 60 ng Color Prestained Protein Standard, Broad Range (11–245 kDa, New-England Biolabs, Frankfurt, Germany). The gel was run at 200 V for 45 min, and afterwards stained with a silver stain kit (Thermo Fisher Scientific) following manufacturer’s instructions. For image acquisition, a ChemiDoc system (Bio-Rad) with Image Lab software 6.1 (Bio-Rad) was used.

## Figures and Tables

**Table 1:** Oligomerization and nitration results of selected samples determined by UV absorbance and mass spectrometry (MS) exemplarily for one independent experiment.

	UV				MS
	DD (%)	DiTyr	ND${}_{\mathrm{tot}}$ (%)	NTyr	NTyr
O_3_/NO_2_ (50/50 ppb), 10 h	2.0	0.2	4.5	0.5	0.4
O_3_/NO_2_ (200/200 ppb), 2 h	2.8	0.3	6.2	0.7	0.7
O_3_/NO_2_ (200/200 ppb), 10 h	5.0	0.6	9.6	1.2	1.1
ONOO^–^/Tyr (1/1)	3.6	0.4	16.8	2.0	1.1
ONOO^–^/Tyr (3/1)	2.4	0.3	21.9	2.6	2.6
ONOO^–^/Tyr (5/1)	1.5	0.2	21.8	2.6	2.7

DD: Dityrosine degree; DiTyr: average number of dityrosine residues calculated per Phl p 5 monomer; ND${}_{\mathrm{tot}}$: total nitration degree determined by RP-HPLC; NTyr: average number of nitrotyrosine residues calculated per Phl p 5 monomer (UV: based on absorbance at 280 and 357 nm; MS: calculated as nitro groups from peak shift).

## Data Availability

The data presented in this study are openly available in an Edmond Repository at https://dx.doi.org/10.17617/3.6f, accessed on 1 June 2021.

## Supplementary Materials

The following are available online at https://www.mdpi.com/article/10.3390/ijms22147616/s1, Figure S1: Exemplary chromatograms from the SEC-DAD analysis of Phl p 5; Figure S2: MALDI spectra of selected samples; Figure S3: Experimental setup used for the exposure of Phl p 5 to O_3_ and NO_2_.

## Abbreviations

The following abbreviations are used in this manuscript:

DiTyr	Dityrosine
DD	Dityrosine degree
MS	Mass spectrometry
ND	Nitration degree
NTyr	Nitrotyrosine
RNS	Reactive nitrogen species
ROI	Reactive oxygen intermediate
ROS	Reactive oxygen species
RP	Reversed-phase chromatography
SEC	Size-exclusion chromatography
Tyr	Tyrosine
