# Supplementary material for: Oligomerization and Nitration of the Grass Pollen Allergen Phl p 5 by Ozone, Nitrogen Dioxide, and Peroxynitrite: Reaction Products, Kinetics, and Health Effects
Source: Int J Mol Sci. 2021 Jul 16;22(14):7616. doi: 10.3390/ijms22147616 (PMC8303544; doi:10.3390/ijms22147616)
Supplement: Supplementary file 1 [file ijms-22-07616-s001.zip › ijms-1264979 supplementary.pdf]

Supplementary Materials: Oligomerization and Nitration of the Grass Pollen Allergen Phl p 5 by Ozone, Nitrogen Dioxide, and Peroxynitrite: Reaction Products, Kinetics, and Health Effects

Anna T. Backes [1,*], Kathrin Reinmuth-Selzle [1], Anna Lena Leifke [1], Kira Ziegler [1], Carola S. Krevert [1,†], Georg Tscheuschner [2], Kurt Lucas [1], Michael G. Weller [2], Thomas Berkemeier [1], Ulrich Pöschl [1], and Janine Fröhlich-Nowoisky [1,*]

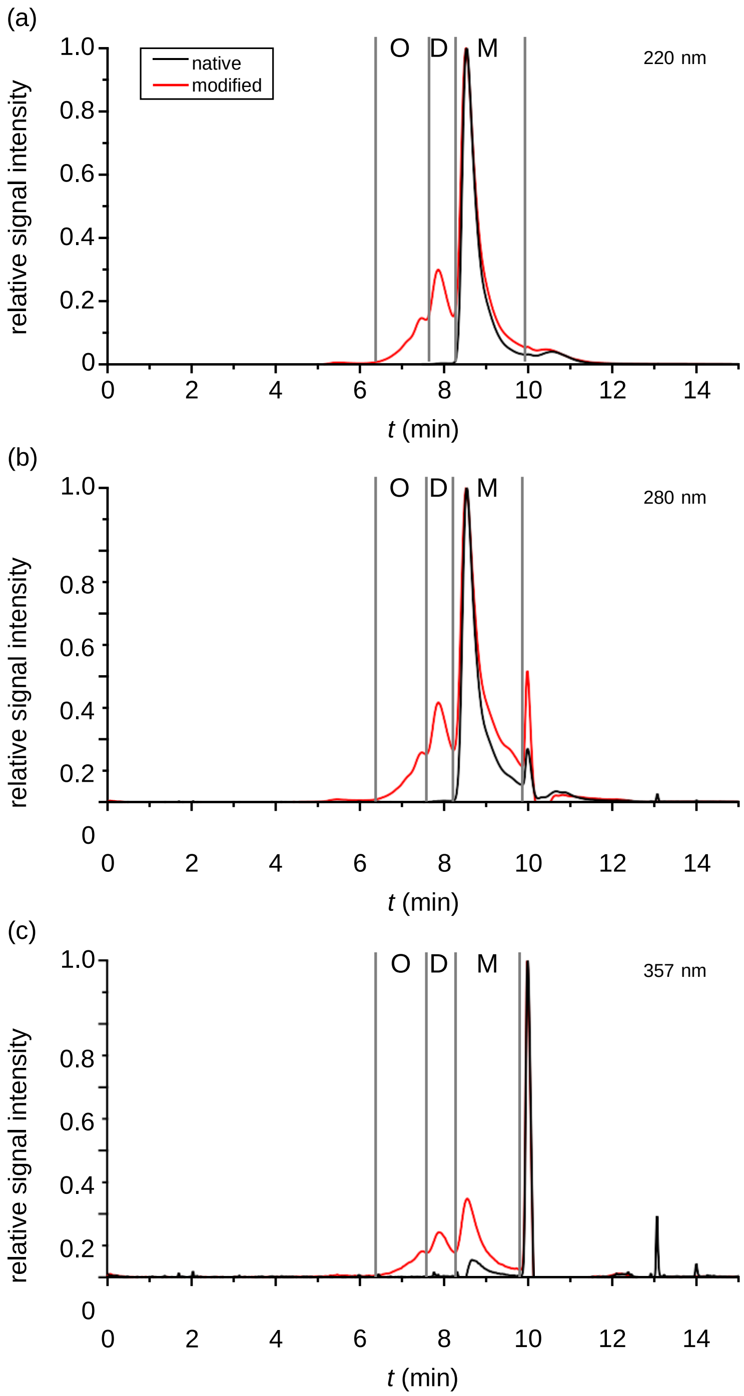

Figure S1. Exemplary chromatograms from the SEC-DAD analysis of Phl p 5 before (black) and after (red) exposure to O_3 and NO_2 measured at different wavelength: (a) 220 nm, (b) 280 nm, (c) 357 nm. Dashed grey lines separate monomer (M), dimer (D), and higher oligomer (O) fractions.

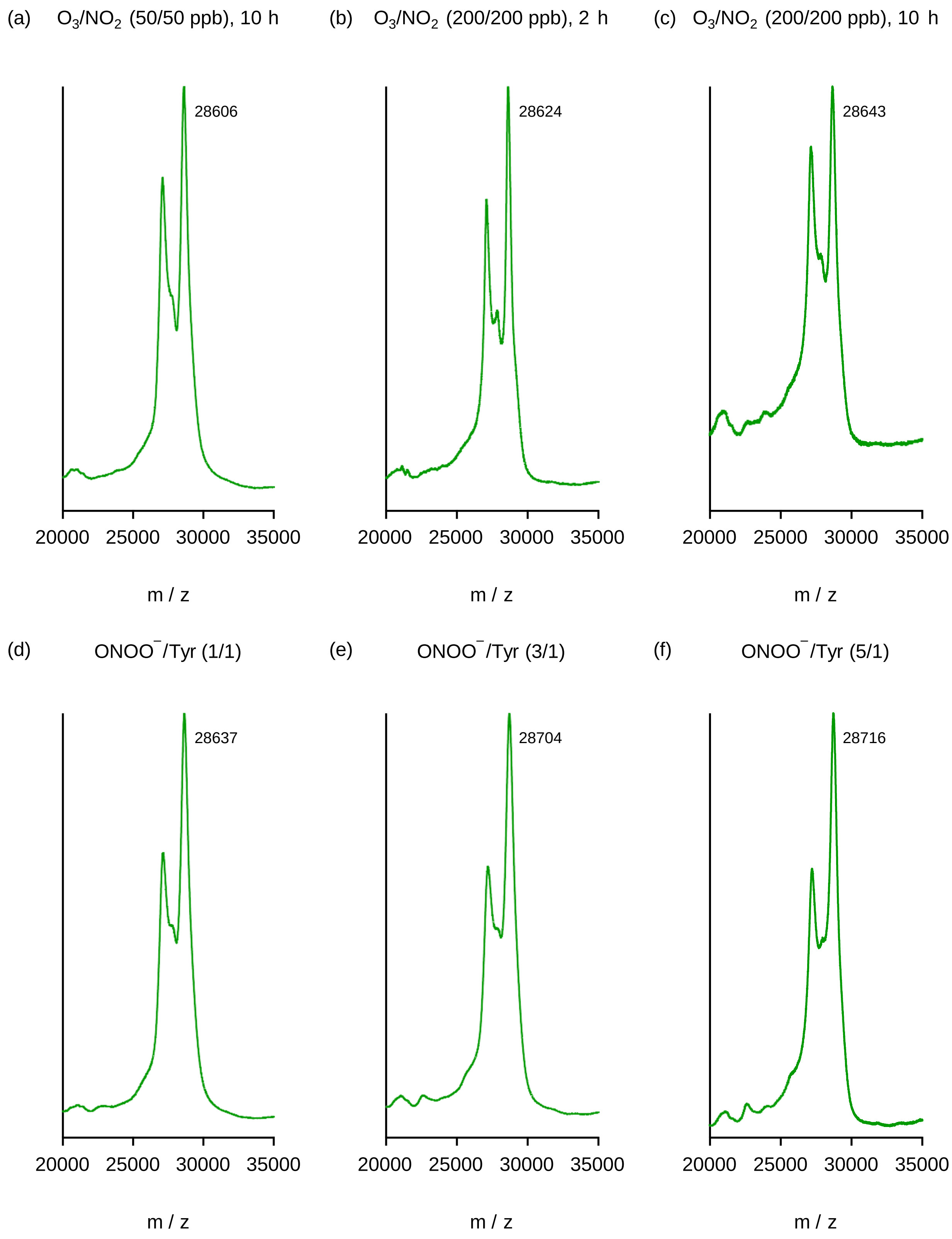

Figure S2. MALDI spectra of selected samples.

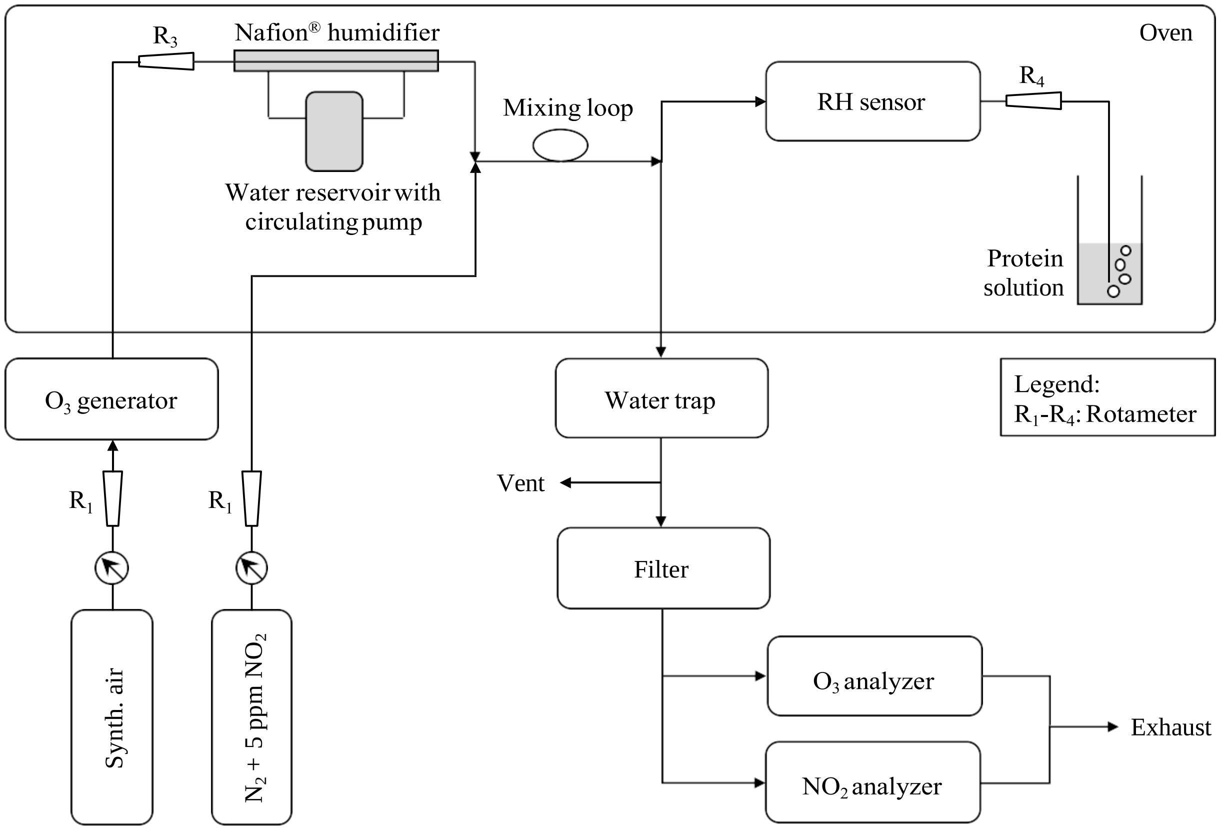

Figure S3. Experimental setup used for the exposure of Phl p 5 to O_3 and NO_2.
